# Risk factors for detection of *Pseudomonas aeruginosa* in clinical samples upon hospital admission

**DOI:** 10.1186/s13756-025-01527-4

**Published:** 2025-02-25

**Authors:** Romeo Reyle, Frank Schwab, Selin Saydan, Michael Behnke, Rasmus Leistner, Petra Gastmeier, Christine Geffers, Tobias Siegfried Kramer

**Affiliations:** 1 Institute of Hygiene and Environmental Medicine, Charité Universitätsmedizin Berlin, Hindenburgdamm 27, 12203 Berlin, Germany; 2 National Reference Center for the Surveillance of Nosocomial Infections, 12203 Berlin, Germany; 3 LADR der Laborverbund Dr. Kramer & Kollegen, 21502 Geesthacht, Germany; 4 Department of Gastroenterology, Infectious Diseases and Rheumatology, Charité Universitätsmedizin Berlin, Hindenburgdamm 30, 12203 Berlin, Germany

**Keywords:** Empirical antimicrobial therapy, Antimicrobial stewardship, Risk factor, Pseudomonas aeruginosa, Antipseudomonal coverage, Watch list, Reserve list

## Abstract

**Background/introduction:**

Antipseudomonal antibiotics are frequently used in patients admitted to hospitals. Many of these substances are classified as a reserve or watch status by the WHO. Inappropriate risk assessment of invasive detection of *P. aeruginosa* (PAE) can be a reason for overuse of antipseudomonal antibiotics. Therefore it is important to define relevant and specific risk factors for invasive PAE detection.

**Objective:**

The objective of this study was to identify risk factors for invasive detection of PAE in patients upon hospital admission.

**Methods:**

All patients 18 years of age and older with a detection of PAE and/or Enterobacterales in clinical samples taken within 48 h of admission to one of the hospitals of Charité Universitätsmedizin Berlin between 2015 and 2020 were included into this retrospective cohort study.

**Results:**

Overall, we included a total of 27,710 patients. In 3,764 (13.6%) patients PAE was detected in clinical samples taken within 48 h after admission. The most frequently detected Enterobacterales was *E. coli* in 14.142 (51%) patients followed by *Klebsiella spp*. in 4.432 (16%) patients. Multivariable regression analysis identified that prior colonisation with a multi drug resistant PAE or detection of a PAE in clinical samples during a previous hospitalisation increased the risk for invasive detection of PAE (OR 39.41; 95% CI 28.54–54.39) and OR 7.87 (95% CI 6.60–9.38) respectively. Admission to a specialised ward for patients with cystic fibrosis was associated with an increased risk (OR 26.99; 95% CI 20.48–35.54). Presence of chronic pulmonary disease (OR 2.05; 95% CI 1.85–2.26), hemiplegia (OR 2.16; 95% CI 1.90–2.45) and male gender (OR 1.60; 95% CI 1.46–1.75) were associated with a modest increase in risk for presence of PAE.

**Conclusion:**

Patients with a prior detection of *P. aeruginosa* or admission to a cystic fibrosis ward had the highest risk for invasive detection of *P. aeruginosa.* Adherence to specific risk scores based on local risk factors could help to optimize prescription of anti-pseudomonal antibiotics that categorized as reserve and watch.

## Introduction

The increase of antimicrobial resistance is an essential threat to human healthcare [1]. Cassini et al. estimate that more than 33,110 deaths in Europe are caused by multidrug resistant pathogens [2]. In Germany an increase of healthcare associated infection with multidrug resistant gram negative pathogens is observable [3]. Antimicrobial Stewardship is one of the most important strategies against antimicrobial resistance [4, 5]. Improved and rational use of antimicrobial substances could lower resistance rates [6]. In Europe most antibiotics in hospitals are prescribed for community acquired infections with a third of prescriptions potentially covering *Pseudomonas aeruginosa* (PAE) [7]. In German hospitals antibiotics with anti-pseudomonal coverage are also frequently used in patients with community and healthcare associated infections [8, 9]. Substances such as Piperacillin-Tazobactam, Meropenem and Ciprofloxacin account for a quarter of all prescriptions. In its aWaRe classification the WHO has categorised most of antipseudomonal antibiotics to the watch or reserve group [10]. An update of EUCAST criteria in 2020 regarding the meaning of the category „ Susceptible, increased Exposure“ might have further increased the use of these substances [11].

De-escalation of antimicrobial therapies is an essential and recommended tool of antimicrobial stewardship [12]. It is safe and effective even in cases with an increased risk for PAE lower respiratory tract infection [13] and suspected sepsis [14]. However, de-escalation is only performed in a fraction of antibiotic prescriptions [9, 15].

Despite the urgent need to improve prescription quality for antipseudomonal antibiotics, only little is known about the reasons for prescriptions of these broad-spectrum antibiotics.

In guidelines risk factors for PAE as a potential cause of lower respiratory tract infections are frequently mentioned, but infrequently reported and or weighted [16, 17]. For other types of infections, only few studies exist for specific patient populations [18, 19].

Therefore, we aim to identify risk factors for detection of PAE in clinical samples acquired within 48 h after hospital admission that are easy asses. In order to potentially improve selection of antipseudomonal antibiotics in this setting.

## Methods

### Participants

We identified all patients 18 years and older that were admitted to one of the three hospitals of Charité Universitätsmedizin Berlinwhich has all together account for about 3000 beds. Prior to the start of the study, we obtained a positive vote from the Charité Ethics Board (internal processing key EA1/264/21). This investigation was then performed as a retrospective cohort study. Cases were identifiedfrom the Institutions own database. We included all cases in patients 18 years of age or older with a finding of PAE and/or Enterobacterales in clinical samples obtained within 48 h of admission. Samples were stratified according to their collection site. In case of multiple samples from a single patient, the most invasive location was chosen.

Cases were defined as patients with the first invasive detection of PAE in a sample taken within 48 h of admission during the study period. Patients that had both invasive PAE and Enterobacterales were accounted to the PAE group. Colonization with PAE was defined as any detection of PAE in the patient during the 12 months prior to admission. Mortality was assessed based on discharge alive or in-hospital death.

For all of the patients enrolled, the following clinical and demographic characteristics were collected: age, sex, in-hospital death, length of hospital stay (LOS); type of initial ward admitted to, specialty of ward admitted to, stay on an intensive care unit (days)previous detection of any PAE prior to the admission and known colonisation or previous infection with multidrug resistant gram negative rod bacteria. Length of stay in total were defined as length of stay until death or discharge. The Charlson comorbidity index (CCI) was obtained based on the patients’ diagnosed comorbidities while using the method of Charlson et al. [20] and the adaptation for the ICD-10 by Thygesen et al. [21].

### Microbiological methods

Microbiological sampling was performed at the treating physician’s discretion. The samples were at least cultured for forty-eight hours. MALDI TOF MS^®^ and Vitek 2^®^ automated system (Biomerieux, Marcy l’etoile, France) were used for identification and susceptibility testing of bacterial strains. Interpretation of susceptibility happened according to the updated EUCAST definitions at each point in time. Gram negative rods with non-intrinsic resistance against 3rd generation cephalosporins, fluorchinolone and/or carbapenems were defined as MDRGN according to the national recommendations.

### Statistical methods

In the descriptive analysis, the median and the interquartile range (IQR) (25% percentile, 75% percentile) were calculated for continuous parameters and the number and percentage were calculated for binary parameters. Descriptive analysis was performed for the total cohort of patients with positive clinical sample caused by gram-negative pathogens within 48 h after hospital admission and stratified by the presence of PAE or not. Differences were tested using Wilcoxon rank-sum test for continuous variables or Chi-square test for binary variables respectively.

The following patient-based parameters were considered in the analysis: age, gender (male/female) and comorbidities. In addition, the following non-patient-based parameters were used in the analysis:

- the type of unit, where the clinical sample was taken (ICU, IMC, regular ward; specialization).
- the type of clinical sample (subgroup).
- the year and the season, when the clinical sample was taken and.
- detection of *Pseudomonas aeruginosa* in clinical samples prior to the admission.
- colonization with multidrug resistant gram negative bacteria (MDRGN) before or simultaneously to the time point the clinical sample was taken.

Multivariable logistic regression analysis was performed to identify independent risk factors and confounders for the outcome “presence of PAE” in the positive clinical sample within 48 h after admission in the hospital. The multivariable model building strategy was performed in a stepwise backward approach. From a full model including all investigated parameters, non-significant parameters were removed with the significance level of *p* = 0.05 and above.

P-values less than 0.05 were considered significant. All analyses were exploratory in nature were performed using SPSS version 29 (IBM SPSS statistics, Somer, NY, USA) and SAS version 9.4 (SAS Institute, Cary, NC, USA).

## Results

**Fig. 1:**
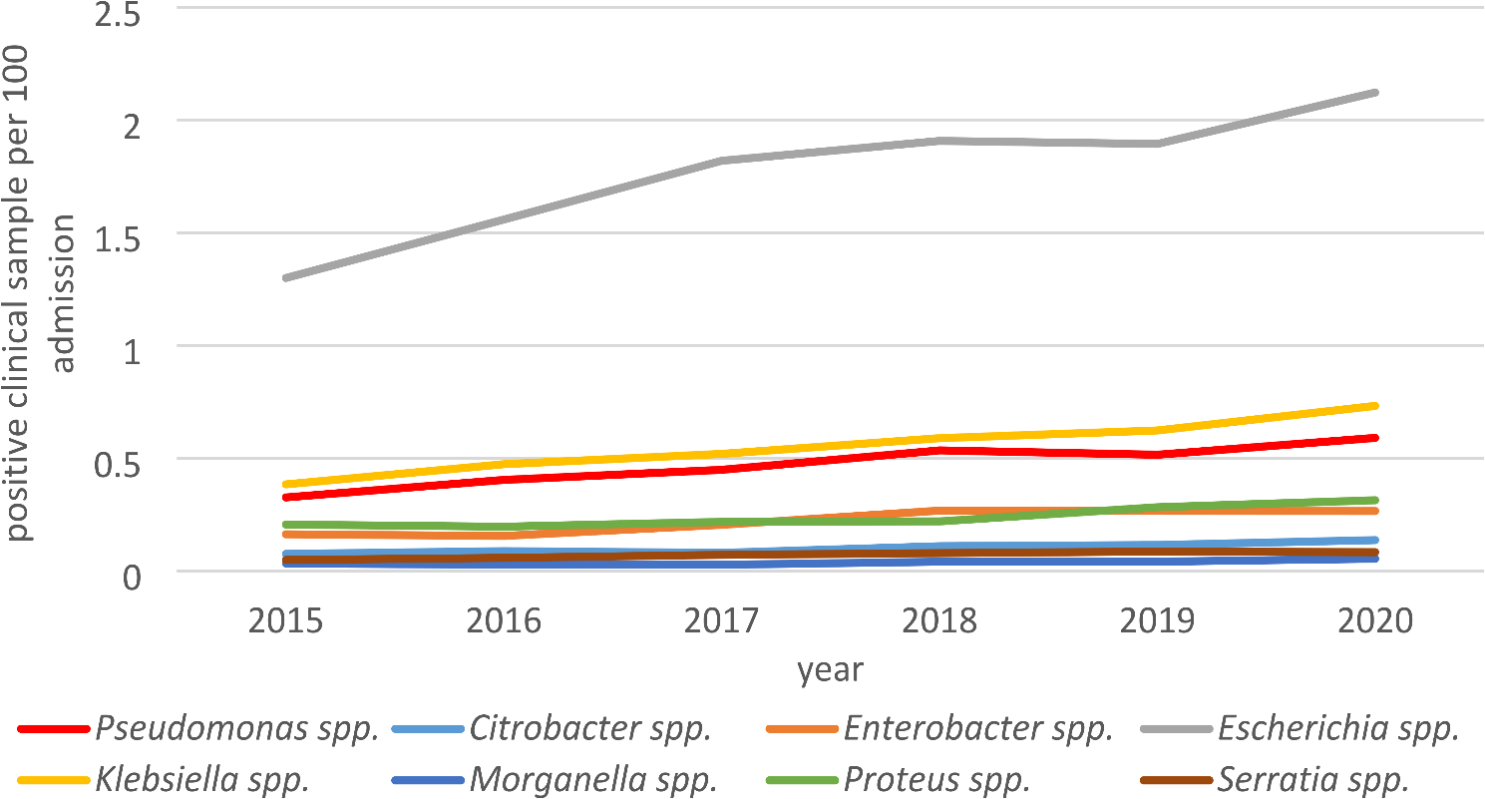
Yearly development of admitted cases with positive sample with GN-pathogens within the first 48 h from the admission to the hospital

Of the 802,859 cases admitted to the hospital between 2015 and 2020 a total of 27,710 (3.45%) admitted cases had clinical samples with growth of GN-pathogens within 48 h from admission (Table 1). A total of 3764 (13.6%) patients had growth of PAE in a clinical sample. However, E. *coli* was the GN 14,142 (51%) most frequently detected followed by *Klebsiella spp.* 4432 (16%). Even though the frequency of GN-pathogens in clinical samples increased over time, their sequence did not (Fig. 1).

**Table 1:** Patients (*N* = 27,710) with positive clinical sample with Gram negative-pathogen during the first 48 h from admission

Parameter	Total amount (%)/median (IQR)
Admissions	27,710 (100%)
Year 2015	3413 (12.3%)
Year 2016	4172 (15.1%)
Year 2017	4170 (15%)
Year 2018	5111 (18.4%)
Year 2019	5542 (20%)
Year 2020	5302 (19.1%)
Length of stay	7 (4–14)
Age	67 (53–77)
Gender female	14,422 (52%)
Gender male	13,288 (48%)
Comorbidities	
Charlson Comorbidity Score	5 (2–8)
Myocardial infarction	558 (2%)
Chronic heart failure	3981 (14.4%)
Peripheral vascular disease	2111 (7.6%)
Cerebrovascular disease	2555 (9.2%)
Dementia	1438 (5.2%)
Chronic pulmonary disease	5573 (20.1%)
Rheumatic disease	1056 (3.8%)
Peptic ulcer disease	357 (1.3%)
Mild liver disease	1623 (5.9%)
Diabetes without chronic complications	4770 (17.2%)
Diabetes with chronic complications	1240 (4.5%)
Chronic kidney disease	10,333 (37.3%)
Malignant tumor	4911 (17.7%)
Moderate to severe liver disease	834 (3%)
Metastasized solid tumor	3032 (10.9%)
AIDS HIV	116 (0.4%)
Hemiplegia	2417 (8.7%)
Leukaemia	453 (1.6%)
Lymphoma	933 (3.4%)
Type of Unit	
Intensive care unit (ICU)	4884 (17.6%)
Regular ward (RW) & intermediate care unit(IMC)	22,826 (82.4%)
Interdisciplinary	4751 (17.1%)
Neurology / Neurosurgery	1606 (5.8%)
Visceral surgery	2600 (9.4%)
Internal medicine	5257 (19%)
Nephrology	3226 (11.6%)
Pediatrics	888 (3.2%)
Oncology	1908 (6.9%)
Obstetrics & Gynaecology	1238 (4.5%)
Urology	1670 (6%)
Cardiology / Visceral surgery	2876 (10.4%)
Cystic fibrosis	994 (3.6%)
Infectious Diseases	1720 (6.2%)
Anaesthesiology	1481 (5.3%)
other	9366 (33.8%)
Clinical Material	
Blood culture	3112 (11.2%)
Gastrointestinal tract	1246 (4.5%)
CSF/otolaryngologic	396 (1.4%)
Respiratory Tract	3255 (11.7%)
Skeletal	233 (0.8%)
others	5703 (20.6%)
urine	13,765 (49.7%)
GN pathogen species	
*Citrobacter spp.*	811 (2.9%)
*Enterobacter spp.*	1769 (6.4%)
*E. coli*	14,142 (51%)
*Klebsiella spp.*	4432 (16%)
*Morganella spp.*	301 (1.1%)
*P. aeruginosa*	3764 (13.6%)
*Proteus spp.*	1919 (6.9%)
*Serratia spp*.	572 (2.1%)
Infection & MDR detection prior to invasive detection	
Previous invasive *P. aeruginosa*	1071 (3.9%)
Colonization MDR *P. aeruginosa*	630 (2.3%)
Colonization MDR *E. coli*	1662 (6%)
Colonization MDR *Klebsiella spp.*	599 (2.2%)
Colonization MDR *Enterobacter spp.*	90 (0.3%)
Colonization other MDRGN	142 (0.5%)

Patients with growth of GN-pathogens in clinical samples had a median age of 67 (IQR:53–77). They had a median CCS of 5 (IQR:2–8). The Univariable logistic regression identified several potential risk factors for the “presence of PAE” in clinical samples (Table 2). Only for those deemed relevant and statitically significant multivariable analysis was performed.

**Table 2:** Results of univariable analysis: admissions (*N* = 27,710) with positive clinical sample with Gram negative-pathogen during the first 48 h from admission stratified by “presence of PAE” or not

Parameter	*P*. aeruginosa			*p*-value
	no	yes	% with *P. aeruginosa*	
	Total number (%)/median (IQR)	Total number (%)/median (IQR)		
Admissions	23,946 (100%)	3764 (100%)	13.6%	
Year 2015	2974 (12.4%)	439 (11.7%)	12.9%	0.534
Year 2016	3603 (15%)	569 (15.1%)	13.6%	
Year 2017	3618 (15.1%)	552 (14.7%)	13.2%	
Year 2018	4382 (18.3%)	729 (19.4%)	14.3%	
Year 2019	4795 (20%)	747 (19.8%)	13.5%	
Year 2020	4574 (19.1%)	728 (19.3%)	13.7%	
Length of Stay	7 (4–14)	9 (5–16)		< 0.001
Age	68 (54–77)	64 (47–76)		< 0.001
Gender male	12,919 (54%)	1503 (39.9%)	10.4%	< 0.001
Gender female	11,027 (46%)	2261 (60.1%)	17.0%	
Comorbidities				
Charlson Comorbidity Score	5 (2–8)	4 (2–7)		< 0.001
Myocardial infarction	517 (2.2%)	41 (1.1%)	7.3%	< 0.001
Chronic heart failure	3579 (14.9%)	402 (10.7%)	10.1%	< 0.001
Peripheral vascular disease	1906 (8%)	205 (5.4%)	9.7%	< 0.001
Cerebrovascular disease	2145 (9%)	410 (10.9%)	16.0%	< 0.001
Dementia	1315 (5.5%)	123 (3.3%)	8.6%	< 0.001
Chronic pulmonary disease	3652 (15.3%)	1921 (51%)	34.5%	< 0.001
Rheumatic disease	968 (4%)	88 (2.3%)	8.3%	< 0.001
Peptic ulcer disease	311 (1.3%)	46 (1.2%)	12.9%	0.698
Mild liver disease	1274 (5.3%)	349 (9.3%)	21.5%	< 0.001
Diabetes without chronic complications	4254 (17.8%)	516 (13.7%)	10.8%	< 0.001
Diabetes with chronic complications	1131 (4.7%)	109 (2.9%)	8.8%	< 0.001
Chronic kidney disease	9290 (38.8%)	1043 (27.7%)	10.1%	< 0.001
Malignant tumor	4425 (18.5%)	486 (12.9%)	9.9%	< 0.001
Moderate to severe liver disease	722 (3%)	112 (3%)	13.4%	0.895
Metastasized solid tumor	2767 (11.6%)	265 (7%)	8.7%	< 0.001
AIDS HIV	104 (0.4%)	12 (0.3%)	10.3%	0.308
Hemiplegia	1812 (7.6%)	605 (16.1%)	25.0%	< 0.001
Leukaemia	389 (1.6%)	64 (1.7%)	14.1%	0.733
Lymphoma	863 (3.6%)	70 (1.9%)	7.5%	< 0.001
Type of Unit				
Intensive care unit (ICU)	4064 (17%)	820 (21.8%)	16.8%	< 0.001
Regular ward (RW) & intermediate care unit (IMC)	19,882 (83%)	2944 (78.2%)	12.9%	< 0.001
Interdisciplinary	4382 (18.3%)	369 (9.8%)	7.8%	< 0.001
Neurology / Neurosurgery	1387 (5.8%)	219 (5.8%)	13.6%	0.949
Visceral surgery	2353 (9.8%)	247 (6.6%)	9.5%	< 0.001
Internal medicine	4629 (19.3%)	628 (16.7%)	11.9%	< 0.001
Nephrology	2901 (12.1%)	325 (8.6%)	10.1%	< 0.001
Paediatrics	801 (3.3%)	87 (2.3%)	9.8%	< 0.001
Oncology	1677 (7%)	231 (6.1%)	12.1%	0.051
Obstetrics & Gynaecology	1187 (5%)	51 (1.4%)	4.1%	< 0.001
Urology	1536 (6.4%)	134 (3.6%)	8.0%	< 0.001
Cardiology / Visceral surgery	2645 (11%)	231 (6.1%)	8.0%	< 0.001
Cystic fibrosis	90 (0.4%)	904 (24%)	90.9%	< 0.001
Infectious Diseases	1483 (6.2%)	237 (6.3%)	13.8%	0.807
Anaesthesiology	1257 (5.2%)	224 (6%)	15.1%	0.075
other	8231 (34.4%)	1135 (30.2%)	12.1%	< 0.001
Type of clinical material				
Respiratory tract	1839 (7.7%)	1416 (37.6%)	43.5%	< 0.001
Blood culture	2936 (12.3%)	176 (4.7%)	5.7%	< 0.001
Urine	12,855 (53.7%)	910 (24.2%)	6.6%	< 0.001
Skeletal	216 (0.9%)	17 (0.5%)	7.3%	0.005
Gastro intestinal tract	1142 (4.8%)	104 (2.8%)	8.3%	< 0.001
Central nervous system; otorhinolaryngology	358 (1.5%)	38 (1%)	9.6%	0.020
other	4600 (19.2%)	1103 (29.3%)	19.3%	< 0.001
Infection & MDRGN colonization				
Previous invasive *P. aeruginosa*	319 (1.3%)	752 (20%)	70.2%	< 0.001
Colonization MDR *P. aeruginosa*	51 (0.2%)	579 (15.4%)	91.9%	< 0.001
Colonization MDR *E. coli*	1541 (6.4%)	121 (3.2%)	7.3%	< 0.001
Colonization MDR *Klebsiella spp.*	500 (2.1%)	99 (2.6%)	16.5%	0.033
Colonization MDR *Enterobacter spp.*	81 (0.3%)	9 (0.2%)	10.0%	0.320
Colonization other MDRGN	110 (0.5%)	32 (0.9%)	22.5%	0.002

Multivariable logistic regression analysis identified a variety of independent risk factors for the outcome “presence of PAE” in clinical samples taken within the first 48 h from admission (Table 3).

**Table 3:** Results of multivariable logistic regression analysis for the outcome “presence *of P. aeruginosa*” in clinical sample with Gram negative-pathogen during the first 48 h from admission

Parameter	OR	95%CI	*p*-value
male	1.599	1.461–1.75	< 0.0001
Comorbidities			
Myocardial infarction	0.806	0.706–0.921	0.0015
Chronic heart failure	0.803	0.678–0.952	0.0114
Chronic pulmonary disease	2.045	1.847–2.263	< 0.0001
Mild liver disease	0.686	0.556–0.847	0.0005
Diabetes with chronic complications	0.716	0.567–0.903	0.0048
Chronic kidney disease	0.905	0.82–0.997	0.044
Metastasized solid tumor	0.847	0.727–0.988	0.034
Infection	1.171	1.049–1.306	0.0048
Pneumonia	1.349	1.203–1.513	< 0.0001
Hemiplegia	2.157	1.897–2.453	< 0.0001
Leukaemia	1.524	1.117–2.079	0.0079
Lymphoma	0.715	0.538–0.95	0.0208
Type of ward			
Interdisciplinary	0.742	0.642–0.856	< 0.0001
Neurology / Neurosurgery	1.256	1.049–1.504	0.0129
Internal medicine	1.347	1.191–1.523	< 0.0001
Oncology	1.739	1.446–2.092	< 0.0001
Cardiology Visceral Surgery	0.712	0.598–0.848	0.0001
Cystic fibrosis	26.978	20.481–35.537	< 0.0001
others	1.401	1.256–1.564	< 0.0001
Type of clinical sample			
other	1.338	1.106–1.618	0.0027
Respiratory tract	1.534	1.24–1.898	< 0.0001
Blood culture	0.419	0.328–0.534	< 0.0001
Urine	0.525	0.433–0.638	< 0.0001
Infection & MDRGN colonization			
Previous invasive *P. aeruginosa*	7.869	6.601–9.381	< 0.0001
Colonization MDR *P. aeruginosa*	39.401	28.542–54.391	< 0.0001
Colonization MDR *E. coli*	0.634	0.471–0.854	0.0027
Colonization MDR *Klebsiella spp.*	0.33	0.256–0.425	< 0.0001
Colonization MDR *Enterobacter spp.*	0.537	0.301–0.959	0.0355

Male gender of patients increased the risk for presence of PAE OR 1.60 (95% CI 1.46–1.75). Prior colonisation with a multi drug resistant PAE or detection of a PAE in clinical samples during a previous hospitalisation increased the risk OR 39.41 (95% CI 28.54–54.39) and OR 7.869 (95% CI 6.60–9.38) respectively.

In addition, admission to a cystic fibrosis ward was associated with an increased risk of OR 26.99 (95% CI 20.48–35.54). Admission to other types of wards such as oncology, internal medicine, neurology and others were only associated with a modest but statistically significant increased odds.

Presence of chronic pulmonary disease OR 2.05 (95% CI 1.85–2.26), hemiplegia OR 2.16 (95% CI1.90-2.45), leukaemia OR 1.52 (95% CI 1.12–2.08), as well as the diagnosis of pneumonia OR 1.35 (95% CI 1.20–1.51) and infection OR 1.17 (95% CI 1.05–1.31) were associated with a modest increase in risk for presence of PAE.

Growth of GN-pathogens in samples derived from the lower respiratory tract and other sites were associated with a modest increased risk for detection of PAE with OR 1.53 (95% CI 1.24–1.90) and OR 1.34 (95% CI 1.11–1.62) respectively.

Interestingly enough the detection GN-pathogens in blood cultures and urine were associated with a statistically significant decreased risk for detection of PAE OR 0.42 (95% CI 0.33–0.53) and OR 0.53 (95% CI 0.43–0.67).

In addition, we identified that patients that were colonized with MDRGN Enterobacterales prior to admission, had a decreased risk for invasive detection of PAE.

## Discussion

In this retrospective single centre cohort study, we were able to identify novel as well as well-established risk factors for the presence of PAE in clinical samples acquired immediately after admission. Our findings could potentially support physicians in evaluation if antipseudomonal coverage in calculated antimicrobial therapy is necessary.

Previous infection with PAE as well as colonisation are well known and internationally established risk factors for subsequent or re- infections with PAE [22]. This is especially true for bacterial infections of the lower respiratory tract such as pneumonia [20].

Patients with structural pulmonary disease have an increased risk for bacterial colonisation and infections with PAE [22]. This is based as much on the medical condition itself, as on the recurrent medical treatment for infection and exacerbation often times with antibiotics [23]. Similar observations are true for patients with hemiplegia. Those patients often spend prolonged durations of time in hospitals and rehabilitation facilities and potentially encounter repeated exposition to antibiotics as well as PAE.

In patients with cystic fibrosis the course of disease is in direct correlation to the presence and infection with PAE in the lung [24]. Cystic fibrosis is a known risk factor for PAE when compared to patients without. Due to this fact, studies investigating risk factors for PAE frequently exclude patients with cystic fibrosis in their analysis. However, we tried to achieve a full overview of risk factor for our setting. Only 994 adult patients (3.6%) of the cohort were admitted to the cystic fibrosis ward. Of whom 92 did not have PAE detected in their clinical samples.

In general, PAE plays an important role in LRTI. The detection in microbiological samples derived from other sites is less common and potentially associated with underlying immunosuppressive medical conditions [18]. Our data underline this fact with an increased independent risk for PAE if the sample is derived from the LRT and an independent risk reduction if samples such as blood cultures or even urine grew Gram-negative rods [25]. So, in alternative sites of infection outside the respiratory tract, PAE is less likely to be the cause of infection [26].

Colonization with MDRGN Enterobacterales was associated with significantly decreased risk for detection of PAE in clinical samples with Gram-negative rods upon admission. Underlying reasons could be based on differences ofcharacteristics in patients underlying conditions. Patients colonized with MDR *E. coli* are frequently less impaired and have a lower Charlson Comorbity Score compared to other Enterobacterales. Patients colonized with non-fermentative bacteria are known to even have more severe impairment and higher CharlsonComorbidity scores. In addition, prior antibiotic exposure, and differences in the susceptibility of pathogens could play an important role as well [27]. CRE are relatively rare in Germany. However, nonspecific carbapenem resistance in PAE are the most frequently identified Carbapenem-resistant organisms [28].

There are certain limitations that apply to this study. (i) The retrospective design of this study and the non-clinical definition for presence of PAE in clinical samples potentially affects our findings to a certain degree. Often patients that are colonized with MDRGN are believed to be similar to patients with predetected colonisation or infection with PAE However, in clinical medicine often the presence of a pathogen in a clinical sample is considered for the antibiotic treatment. (ii) Our findings might not be applicable for some other centers, since this cohort is derived from a single university hospital that consist of three independent hospitals and provides specialized care as including patient with cystic fibrosis as well as organ transplant. (iii) We were not able to differentiate between community acquired and healthcare associated infections with readmission. But often this not possible for the physicians evaluating the patients upon admission. iiii) Some of the variables used in this study rather represent surrogates than the directly measured information. iiiii) The definition used for MDRGN is specific to the German healthcare system and unfortunately cannot easily be extrapolated to international definitions.

## Conclusion

*PAE* is frequently cultured from clinical samples taken from patients within 48 h after admission. The overall most important risk factors are prior detection or cystic fibrosis. Knowledge of these risk factors upon admission can significantly help shaping the use of anti-pseudomonal antibiotics. Future studies should investigate the potential of such risk factor analysis in clinical routine for antibiotic prescriptions.

## Data Availability

No datasets were generated or analysed during the current study.
